# 541 Needs Assessment among Adult Burn Survivors’ Caregivers in Korea

**DOI:** 10.1093/jbcr/irad045.138

**Published:** 2023-05-15

**Authors:** Se-Hee Hwang, So-Young Park

**Affiliations:** Hangang Sacred Heart Hospital of Hallym University Medical Center, SEOUL, Seoul-t'ukpyolsi; Ewha Womans University Ewha Institute for Age Integration Research, SEOUL, Seoul-t'ukpyolsi; Hangang Sacred Heart Hospital of Hallym University Medical Center, SEOUL, Seoul-t'ukpyolsi; Ewha Womans University Ewha Institute for Age Integration Research, SEOUL, Seoul-t'ukpyolsi

## Abstract

**Introduction:**

Burn injuries have negative impact on both burn survivors and their loved ones. As family members usually care for burn survivors, it is very critical to understand the concerns of family caregivers and their needs for social services during the process of recovery. The purpose of this study is to assess 1) a broad range of difficulties among family caregivers of adult burn survivors, 2) the frequency of use of social service programs and satisfaction with these services, and 3) the needs of caregivers of burn survivors on the service provision.

**Methods:**

We used a cross-sectional survey design with purpose sampling. We conducted both online and face-to-face surveys from November 2021 to February 2022. A total of 92 family caregivers aged 20 years or older participated in the survey. We measured 1) physical (e.g. chronic diseases such as pain and hypertension), psychological (e.g., family conflicts, communication issues with a burn survivor), financial (e.g., medical expenses, living costs), and social & environmental problems (e.g., living environment, education, discrimination or prejudice due to burn scars and disabilities), 2) the use of social service programs and the level of service satisfaction, and 3) the needs of social service programs (e.g., counseling services for PTSD treatment, financial support, and discharge planning). We used SPSS 24.0 for all data analyses.

**Results:**

The most challenging problems were financial matters followed by psychological, and social & environmental ones among study participants. Study respondents used social service programs to resolve their psychological, financial, and discharge related difficulties. Those who used social service programs showed higher satisfaction with an average of 3 out of 5 points. More than 50% of study participants reported that it was necessary to receive social services on psychosocial, financial, and discharge related problems and they were willing to receive these services in future.

**Conclusions:**

These findings imply that it is necessary to develop and provide needs-based social services for caregivers of burn survivors and that the provision of social services should be expanded further.

**Applicability of Research to Practice:**

Findings of this study demonstrate that social service programs can relieve the burden of caregivers and improve the quality of life of both burn survivors and their caregivers.

